# Can Serum Histone H4 Level Be a Biomarker in Ulcerative Colitis?

**DOI:** 10.5152/tjg.2024.22385

**Published:** 2024-01-01

**Authors:** İrfan Küçük, Fatih Özçelik, Yusuf Yazgan, İsmail Yılmaz, Mustafa Kaplan, İdris Yıldırım

**Affiliations:** 1 Department of Gastroenterology, University of Health Sciences Sultan 2. Abdulhamid Han Training and Research Hospital, İstanbul, Turkey; 2 Department of Medical Biochemistry, University of Health Sciences, Ümraniye Training and Research Hospital, İstanbul, Turkey; 3 Department of Pathology, University of Health Sciences Sultan 2. Abdulhamid Han Training and Research Hospital, İstanbul, Turkey; 4 Department of Internal Medicine, University of Health Sciences Sultan 2. Abdulhamid Han Training and Research Hospital, İstanbul, Turkey

**Keywords:** Ulcerative colitis, histone H4, neutrophil

## Abstract

**Background/Aims:**

Histones are a part of neutrophil extracellular trap molecules which were reported to have diagnostic values in some inflammatory diseases. We aimed to evaluate whether serum histone H4 can be a diagnostic and prognostic marker for ulcerative colitis.

**Materials and Methods:**

This case–control study included 58 ulcerative colitis patients (34 males and 24 females) and 45 healthy controls (25 males and 20 females). The Mayo clinical scoring system was used for the clinical and endoscopic features. Truelove–Witt’s method was applied to the histology activity index. The human histone H4 kit was used for the enzyme-linked immunosorbent assay of serum histone H4.

**Results:**

Serum histone H4 was significantly lower in the ulcerative colitis group compared to the control groups [268 (14-1639) vs. 598 (310-2134) ng/L,* P* < .001, respectively]. Among the ulcerative colitis patients, there was no correlation between serum histone H4 and disease extent, Mayo clinical scoring, Mayo endoscopic activity subscoring, histology activity index, inflammatory markers, d-dimer, and leukocyte and neutrophil counts (*r *< 0.20, *P* > .05). Histone H4 levels were not statistically significant between the patients with no medication and those taking 5-aminosalicylate and/or other agents (*P *> .05). The receiver operating characteristic curve analysis revealed that serum histone H4 concentrations had a 0.782 (95%CI: 0.690-0.857,* P < .*001) diagnostic accuracy for ulcerative colitis. The specificity and sensitivity for the cutoff level of ≤364 ng/L were 88.9% and 72.4%, respectively.

**Conclusion:**

Decreased serum histone H4 values may be used as an auxiliary marker in the progression and diagnosis of ulcerative colitis. Further studies are needed to delineate this relationship between clinical and laboratory traits of ulcerative colitis and serum histone H4.

**Main Points**

- Diagnostic strategies with the possibility of therapeutic interventions can be developed if serum histone H4 (HH4) protein levels relate to the clinical and laboratory features of ulcerative colitis.
- Decreased serum HH4 values may be used as an auxiliary marker in the progression and diagnosis of ulcerative colitis (UC).
- Further studies are needed to delineate the relationship between clinical and laboratory traits of UC and serum HH4.
- The current study is the first clinical trial evaluating the accuracy of serum HH4 levels in the diagnosis and prognosis of UC.

## Introduction

Neutrophilic infiltration into the colonic mucosa is the mainstay of the histopathological features in ulcerative colitis (UC).^1,2^ In response to bacterial products, neutrophils secrete extracellular trap molecules (NETs), including DNA fragments, histones, and bactericidal proteins.^3^ Neutrophile extracellular trap molecules bind to the pathogens and confine them to the site where they provide direct contact with antimicrobial proteins.^3^

Histones are highly basic proteins acting as spools around which DNA winds to create structural units called “nucleosomes.” They participate in the cellular innate immune response and exert harmful as well as protective effects.^3,4^ Histones inhibit thrombomodulin and cause microvascular thrombosis in rats.^5,6^ They are classified as H1, H2A, H2B, H3, and H4. All histones have antibacterial properties, and histone H4 (HH4) is the most potent one.^4,7^ Extracellular DNA complexes and histones induce proinflammatory cytokines by increasing toll-like receptor 2, 4, and 9 activities.^8^

In addition to clinical conditions including sepsis, trauma, and venous thromboembolism,^3^ histones and NETs were investigated in the experimental models of inflammatory bowel diseases (IBDs).^9-11^ Neutrophile extracellular traps were investigated in mucosal biopsies of a limited number of IBD patients.^10-14^ Currently, there is a lack of data about the role of HH4 and other NETs in the clinical and laboratory evaluation of UC.

This study aimed to evaluate whether serum HH4 levels of UC patients could serve as a diagnostic and prognostic marker correlating to the clinical, endoscopic, biochemical, and histological features of the disease. Diagnostic strategies with the possibility of therapeutic interventions can be developed if the serum HH4 protein values were related to the clinical and laboratory features of UC.

## Materials and Methods

### Subjects

Fifty-eight patients with UC and 45 healthy controls admitted to the Gastroenterology Department of University of Health Sciences Sultan 2. Abdulhamid Han Training and Research Hospital, İstanbul, Turkey between April 2021 and January 2022 were enrolled in the study. The Ethics Committee of Sancaktepe Prof. İlhan Varank Training and Research Hospital approved the study (141/07.04.2021). Written informed consent was obtained from all participants. Participants with clinical conditions that can affect serum HH4 levels such as sepsis, any malignancies, venous thromboembolism, or trauma were excluded from the study. Participants with severe organ failure, acute or chronic infections, autoimmune diseases, or gut resection were also excluded from the study.

Disease duration, family history of IBD, extra-intestinal complications, medications, and comorbidities were recorded for all participants. Erythrocyte sedimentation rate (ESR), C-reactive protein (CRP), d-dimer, and other biochemical tests were measured. The UC Mayo clinical scoring system (MCS) was used to assess the disease activity, which was scored between 0 and 12. A score of ≤2 was classified as clinical remission and a score of >2 was classified as activation.

The Mayo endoscopic activity subscoring (MEAS) index was used for the endoscopic activation of UC and classified as remission (0), mild (1), moderate (2), and severe colitis (3). Scores of 0 and 1 were recorded as endoscopic remission, and scores of 2 and 3 were recorded as activation. Disease extent (DE) of UC was defined in agreement with the Montreal classification and grouped as remission, proctitis, left-sided colitis, and extensive colitis. The healthy control group included participants who underwent a colonoscopy for indications other than IBDs.

### Histone H4 Measurement

The serum for HH4 was separated from venous blood samples and after centrifugation at 5000 ×*g *for 10 minutes at 30°C, the supernatant serum was stored at (−) 80°C until analysis for 6-9 months. The commercially available Human Histone H4 (HIST1H4A) Bioassay Technology Laboratory Kit (Cat. No. E5420 Hu, Lot:202201004) was used for the ELISA measurement of the serum HH4 (Intra-Assay: CV <8%, Inter-Assay: CV <10%). Measurements were taken by using the ELISA device (BioTek Epoch 2 with the software Gen5 [Version 2.07], BioTek Instruments Inc., Winooski, VT, USA).

### Histopathologic Evaluation

The same pathologist evaluated the formalin-fixed paraffin-embedded H&E-stained colonic biopsies of the UC patients and performed grading through a scale similar to that developed by Truelove and Witt.^15,16^ Active inflammation, chronic inflammation, and crypt distortion were the components of the scale. The histopathologic evaluation with the histology activity index (HAI) was defined as the sum of the scores of these components.

### Statistical Analysis

Statistical analyses were performed using the IBM Statistical Package for Social Sciences (SPSS) statistics version 25.0 (IBM Corp., Armonk, NY, USA) and the MedCalc® statistical software version 15.8 programs. Descriptive statistics were shown using the proportion for categorical variables, whereas the mean with SD and the median with its extremes were used for continuous variables. Comparisons were conducted using the Student’s *t*-test and the Mann–Whitney *U* test for continuous variables and by the chi-square test for categorical variables. The difference among more than 2 groups was sought by the Kruskal–Wallis test. For post hoc multiple comparisons, Dunn’s test was used. Diagnostic accuracy was evaluated using receiver operating characteristics (ROC) curve analysis. Using Pearson’s correlation analysis and Spearman’s correlation analysis, associations were denoted in accordance with the variables’ distributions. The CI for statistical significance was defined as 0.95.

## Results

Fifty-eight patients (34 males and 24 females) with UC and 45 healthy controls (25 males and 20 females) were evaluated in the study. There was no statistically significant difference between the UC patients and the healthy controls in terms of age and gender (*P* > .05). Demographic, clinical, and histopathological characteristics of the study population are presented in Table 1. It was determined that 64% of the patients with UC received 5-aminosalicylate (5-ASA) treatment, while 12% received azathioprine, 10% anti-tumor necrosis factor (TNF) alpha (α), and only 3% steroid treatment.

There were no significant differences between the leukocyte and neutrophil counts of the 2 groups (*P* > .05). Erythrocyte sedimentation rate, CRP, and d-dimer levels in patients with UC were higher, whereas albumin was lower than that of the control group (*P* < .05) (Table 1). In UC patients, significant correlations were found between the neutrophil counts and MCS (*r* = 0.296, *P* = .024) and MEAS (*r* = 0.340, *P* = .009).

**Figure 1.**
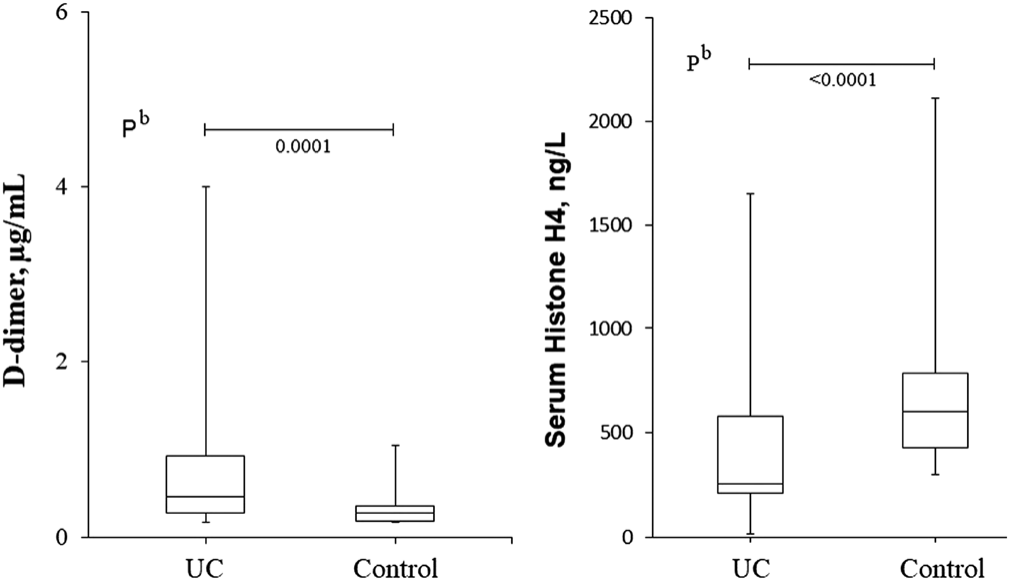
Comparison of d-dimer and serum histone H4 levels of ulcerative colitis patients and the control group.

Serum HH4 concentration was significantly lower in UC patients compared to the control group [268 (14-1639) vs. 598 (310-2134) ng/L,* P < *.001, respectively] (Figure 1). There was no correlation between serum HH4 and DE, MCS, MEAS, HAI, CRP, ESR, d-dimer, leukocyte, and neutrophil counts of the UC patients (*r* < 0.20, *P* > .05). Moderately negative correlations were found between serum HH4 concentrations and CRP and ESR values of all participants (*r = *−0.340, *P* < .001; and *r *= −0.326, *P* < .001, respectively).

Serum HH4 concentrations of UC patients in remission according to the MCS were not statistically significant in comparison with patients with active disease (492 ± 458 and 450 ± 384 ng/L,* P *= .939). Serum HH4 levels of UC patients in remission according to colonoscopy (MEAS:0.1) were not statistically significant compared to patients with active disease (MEAS:2.3) (480 ± 408 and 444 ± 394 ng/L, *P = .*968).

Serum HH4 values between the control group and the treatment groups in UC patients are presented in Table 2. Comparison analyses revealed a significant difference between the control and treatment groups that were the sum of those receiving ASA and/or other agents (*P *< .001). Serum HH4 levels were not significantly different between the patients with no medications and those taking 5-ASA or 5-ASA and/or other agents (*P* > .05).

There was no difference between histone levels according to UC’s MEAS (*P *> .05) (Table 3). It was determined that there was a difference between the values of the control group and those with inactive and active inflammation in terms of active inflammation [Kruskal–Wallis test, 598 (310-2170) vs. 247 (14-803) and 270 (167-1639) ng/L, *P < *.0001]. When the groups were compared with Dunn’s multiple comparison test and while there was a difference between the values of the control group and those with inactive disease, and between the values of the control group and those with active disease (*P* < .05 and* P < *.001, respectively), there was no statistical difference between the values of those with inactive disease and those with active disease (*P* > .05).

**Figure 2.**
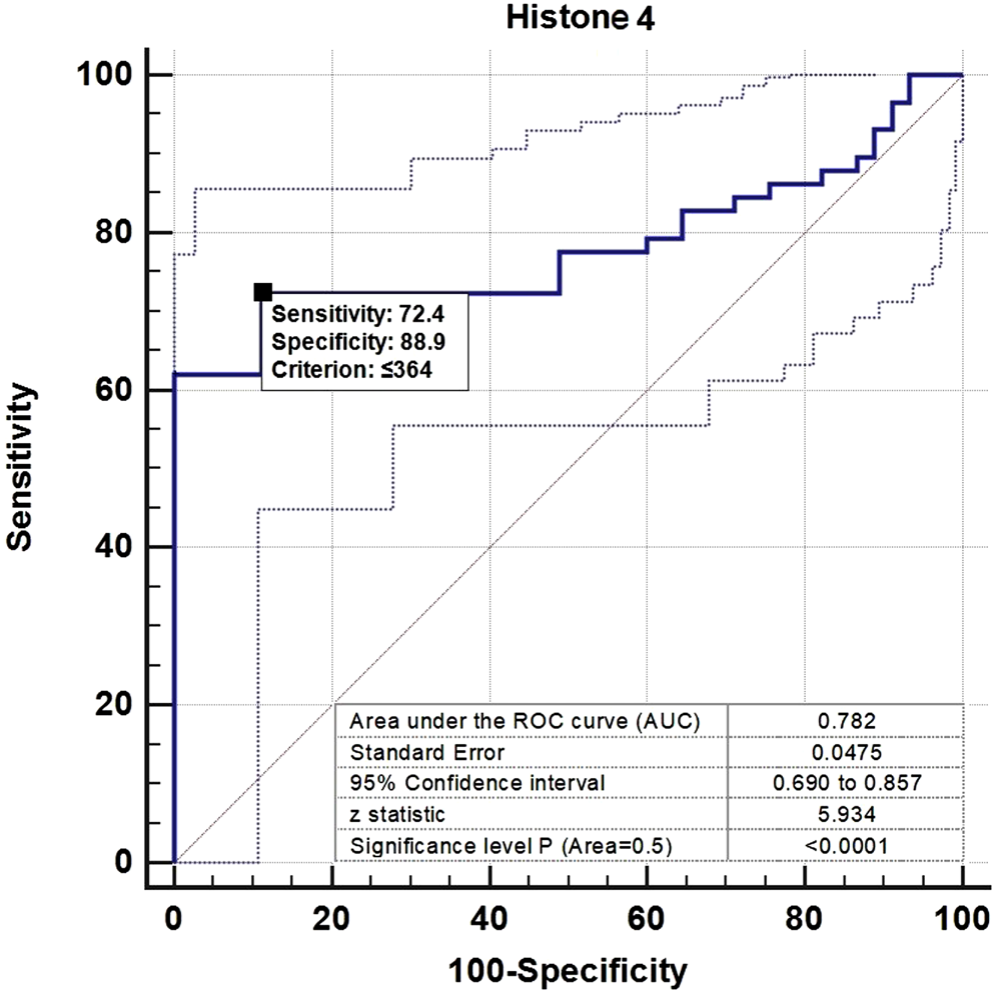
Receiver operating characteristics curve of serum histone H4 for determining the presence of ulcerative colitis.

The ROC curve analysis revealed that serum HH4 concentrations had a 0.782 (95% CI: 0.690-0.857,* P *< .001) diagnostic accuracy for UC. The specificity and sensitivity for the cutoff level of ≤364 ng/L were 88.9% and 72.4%, respectively (Figure 2).

Correlation analyses also revealed moderate and positive correlations between d-dimer and DE, MEAS, and MCS (*r* = 0.392, *P* = .002; *r* = 0.354, *P* = .006; and *r* = 0.394, *P* = .002, respectively).

## Discussion

Exaggerated activation of the neutrophils causes mucosal damage. The severity of inflammation has been found to be associated with the quantity of the neutrophils, but the precise role of neutrophils in IBDs remains controversial.^17,18^ Neutrophil counts were not different in both groups in this study, which can be ascribed to the medications used in UC patients. It is noteworthy that the neutrophil counts correlated positively to the clinical and endoscopic mucosal activity scores. This result supports previous reports regarding the prognostic significance of neutrophils in UC.^17,18^

Neutrophils can eliminate microorganisms by either phagocytosis or NETs formation, the term called NETosis, and it requires the production of reactive oxygen species (ROS) and neutrophil elastase. Histones are the important actors of NETosis.^3,6,19^ The dichotomous choice of neutrophils, phagocytosis, or NETosis depends on the proinflammatory cytokines, lipopolysaccharides, metabolic and adhesive stimuli, or the size of the antigenic particle. Large antigenic structures like parasites can be eliminated by NETosis.^19^

Tsaprouni et al^10^ reported increased HH4 values in the biopsies of rats with the inflamed segments of the bowel and in the biopsies of Crohn’s disease (CD) patients but decreased HH4 values in the biopsies of the uninflamed segments of the bowel in the 2 groups. Köker et al^20^ reported higher serum HH4 values in CD patients than in the control group. These researchers declared serum HH4 as a predictive marker for mucosal healing in CD. Unlike these studies, in this study, UC patients were selected instead of CD patients, and the most important finding is that serum HH4 values were lower in the UC patients compared to the control group.

Although higher serum HH4 levels have been reported due to chronic inflammation in IBD according to the literature,^10,20^ the possible reason for the low HH4 results in patients with UC in this study can be due to the antimicrobial activity of HH4, which is a part of the innate immune system.^21^ Based on this information, it can be considered that the mucosal neutrophils in UC patients cannot produce HH4 adequately and cannot prevent mucosal bacterial invasion; therefore, regional inflammation cannot be limited. Moreover, the fact that histones can directly destroy bacteria, viruses, and parasites as antimicrobial peptides and can reduce harmful effects by regulating inflammatory pathways supports the results of this study.^22^ Again, the H4 levels of the control group were found to be higher than those of patients with UC with inactive and active inflammation, which also supports the above findings.

Özgür et al^23^ declared higher serum HH4 levels in patients with colon adenocarcinoma but lower values in patients with nonneoplastic and precancerous colonic polyps when compared to healthy controls. This result may provide a clue for the different bioactivity of HH4. Although UC and CD belong to the same family, they have their own characteristics. Different activities of the pathogenicity-related proteins can be attributed to the different features of IBDs and this condition may be an explanation for the lower serum HH4 values of UC patients in this study and the higher values reported in CD patients.^20^ Thus, serum HH4 may be proposed as a marker for the differential diagnosis of IBDs.

Dexamethasone suppresses NETosis and anti-TNF-α treatment diminishes the NETs’ formation of circulating neutrophils which are stimulated by TNF-α in UC patients.^24,25^ These findings delineate the effect of treatment on NETosis. In this study, 67.24% of the participants were taking medications including immunosuppressive, biological agents, steroids, and 5-AMA. No statistically significant association was found between the participants without any medication and those undergoing treatment for UC, but the serum HH4 values of the control group were higher than the values in the treatment group of UC patients. No significant difference was reported between the treatment groups.

Although not statistically significant, the mean serum HH4 value of the UC participants without any medication was higher than the mean serum HH4 value of those undergoing treatments. It is thought that medications via inhibiting proinflammatory stimuli in UC might also be responsible for low serum HH4 values. The number of patients receiving steroids, immunosuppressive, and anti-TNF-α agents in this study was smaller in size and larger sample-sized cohorts might present significant results. Köker et al^20^ reported higher serum HH4 values in CD patients than participants in the control group, but the treatment profiles of CD patients were not mentioned.

Correlation is a method of evaluating the possible 2-way linear relationship between 2 continuous variables.^26^ In addition, biological data often do not show a Gaussian distribution and may show high or low levels of clustering associated with the disease. Therefore, it is thought that no correlation could be found between HH4 and CRP and ESR in the UC patient group.

Despite the prothrombotic features of histones,^5,6^ correlations between serum HH4 and d-dimer were not significant, and this result can also be attributed to the deficient HH4 release. d-dimer alone in the UC group correlated positively to DE and endoscopic, clinical severity scores. Plasma d-dimers are markers of thromboembolic events. D-dimer is also an acute phase reactant. The results of this study were consistent with previous reports.^1,4,27^

A study by Theede et al^28^ using the Mayo Score found a positive association between increased clinical disease activity and the degree of fecal calprotectin (FCp) in UC. This finding is likely due to FCp being a strong marker of inflammation. Again, in a recent study,^29^ FCp was found to be related to both active disease and mucosal healing in evaluating the disease activity of UC, although the threshold value could not be accurately determined. In this study, HH4, which is known to stimulate pro-inflammatory cytokines and can be detected in serum, was used to evaluate the clinical disease activity of UC instead of calprotectin, which is abundant in leukocytes. However, the HH4 results were found to be negatively correlated with UC clinical activity. This finding is likely because HH4 has a different biological behavior to CRP and FC in inflammatory events. Because inflammatory cells (such as neutrophils, lymphocytes, macrophages, and plasma cells), which are a strong determinant of inflammation, can cause an excessive inflammatory response and can cause damage to mucosal tissues, as well as accelerate NETosis (via mediators such as HH4) eliminating microorganisms and limiting inflammation.^18-2^ ^0^

It is still unclear whether NETosis is a cause or a result of the inflammation.^25^ Some reports propose NETs as causative factors of IBDs but opposite reports also exist.^30^ Inhibition of NETosis has also been proposed as a therapeutic option in experimental colitis and this can be another focus for future research.

The major limitation of the current study is the small number of the study population especially for UC participants. Larger cohorts might exhibit different results. An assessment of HH4 in colonic biopsy samples in addition to serum HH4 values could reveal precise information for HH4 activity in UC.

The main limitation of the study is that it included a small number of patients who received different treatments, some of which directly affected histone levels. In addition, the determination of serum H4 levels at the time of diagnosis before starting treatment in patients with different activity groups may be a different study.

In conclusion, decreased serum HH4 values may be used as an auxiliary marker in the progression and diagnosis of UC. Whether HH4 values can be used to differentiate CD from UC needs further prospective comparative studies. This study is the first clinical trial about the serum HH4 values in UC and should be considered as a preliminary and first step for future research.

## Figures and Tables

**Table 1.** Demographic, Clinic, and Laboratory Characteristics of the Study Population

Demographic Features	Patients with UC	Controls	*P*
n	58	45	–
Gender, n (%)			
Female	24 (41)	20 (44)	.789^a^
Male	34 (59)	25 (56)	
Age, years			
Mean ± SD	40 ± 14	40 ± 14	.773^a^
Median (minimum–maximum)	38 (18-74)	36 (20-73)	
ESR (mm/h), mean ± SD	41.7 ± 35.2	10.8 ± 9.7	<.001^b^
Albumin (g/dL), median (minimum–maximum)	4.5 (2.1-5.4)	4.6 (3.4-5.3)	.043^a^
Leukocytes (×10^3^/μL), mean ± SD	8.8 ± 3.3	8.0 ± 2.5	.191^b^
CRP (mg/L), median (minimum–maximum)	10.4 (0.2-138)	2.1 (0.2-14.2)	<.001^a^
Neutrophils (×10^3^/μL), mean ± SD	5.6 ± 2.6	5.0 ± 2.0	.184^b^
D-dimer (μg/mL), median (minimum–maximum)	0.46 (0.17-4.00)	0.27 (0.17-1.04)	<.001^a^
Serum histone H4 (ng/L), median (minimum–maximum)	268 (14-1639)	598 (310-2134)	<.001^a^
Disease duration, years			
Mean ± SD	4 ± 4		
Median (minimum–maximum)	2 (0-20)		
Extent of UC, n (%)			
Remission	3 (5.17)		
Proctitis	14 (24.13)		
Left-sided colitis	21 (36.21)		
Extensive colitis	20 (34.48)		
Mayo endoscopic activity score, n (%)			
0 (Inactive disease)	4 (6.90)		
1 (Mild)	19 (32.75)		
2 (Moderate)	21 (36.21)		
3 (Severe)	14 (24.13)		
Treatments, n (%)			
No treatment	19 (32.75)		
5-ASA	37 (63.79)		
Steroids	3 (5.17)		
Azathioprine	7 (12.06)		
Anti-TNF α	6 (10.34)		
Family history of IBD, n (%)	6 (10.24)		
Mayo clinical activity score, median	5.67		
Remission (score ≤ 2), n (%)	12 (20.68)		
Activation (score > 2), n (%)	46 (79.31)		
Histology activity index	5.19 ± 2.30		

^a^Mann–Whitney *U* test;^ b^unpaired *t*-test. Parametric data were shown as mean ± SD and nonparametric data as median (minimum–maximum).

CRP, C-reactive protein; ESR, erythrocyte sedimentation rate; IBD, inflammatory bowel disease; 5-ASA, 5-aminosalicylate; TNF, tumor necrosis factor; UC, ulcerative colitis.

**Table 2.** Comparison of Serum Histone H4 Values in Healthy Controls and Ulcerative Colitis Patients Receiving Different Medications

	Healthy Controls	No Medications	Only 5-ASA	5-ASA and/or the Other Agents	*P*
n	45	19	26	39	–
Serum HH4 (ng/L)	598 (310-2134)	289 (209-1639)	256 (14-1454)	266 (14-1454)	<.001^a,b^

The intergroup comparisons were made when *P*-values of <.05 (significant) were found in the Kruskal–Wallis test.

Comparisons: Controls vs. no medications groups (*P* < .05), controls vs. only 5-ASA groups (*P* < .001), controls vs. 5-ASA and/or the other agent groups (*P* < .001), no medications vs. treatment with only 5-ASA groups (*P* > .05), no medications vs. 5-ASA and/or the other agent groups (*P* > .05).

^a^Kruskal–Wallis test (nonparametric analysis of variance) with post hoc test; ^b^Dunn’s multiple comparison test.

HH4, histone H4; 5-ASA, 5-aminosalicylate.

**Table 3.** Histone H4 Levels According to Ulcerative Colitis Mayo Endoscopic Activity Score

	MEAS-0	MEAS-1	MEAS-2	MEAS-3	*P*
n	5	18	21	14	
Histone H4 (ng/L)	364 (242-803)	258 (14-1454)	247 (167-1639)	353 (183-1365)	.5977^ a^

^a^Kruskal–Wallis test (nonparametric analysis of variance).

MEAS, Mayo endoscopic activity score.
